# Crucial Role of Microbiota in Experimental Psoriasis Revealed by a Gnotobiotic Mouse Model

**DOI:** 10.3389/fmicb.2019.00236

**Published:** 2019-02-21

**Authors:** Zuzana Stehlikova, Klara Kostovcikova, Miloslav Kverka, Pavel Rossmann, Jiri Dvorak, Iva Novosadova, Martin Kostovcik, Stepan Coufal, Dagmar Srutkova, Petra Prochazkova, Tomas Hudcovic, Hana Kozakova, Renata Stepankova, Filip Rob, Katerina Juzlova, Jana Hercogova, Helena Tlaskalova-Hogenova, Zuzana Jiraskova Zakostelska

**Affiliations:** ^1^Institute of Microbiology of the Czech Academy of Sciences, v.v.i., Prague, Czechia; ^2^First Faculty of Medicine, Charles University, Prague, Czechia; ^3^Institute of Molecular Genetics of the Czech Academy of Sciences, v.v.i., Prague, Czechia; ^4^Institute of Experimental Medicine of the Czech Academy of Sciences, v.v.i., Prague, Czechia; ^5^ BIOCEV, Institute of Microbiology, Czech Academy of Sciences, Vestec, Czechia; ^6^ Institute of Microbiology of the Czech Academy of Sciences, v.v.i., Novy Hradek, Czechia; ^7^ Department of Dermatology and Bulovka Hospital, Second Faculty of Medicine, Charles University, Prague, Czechia

**Keywords:** psoriasis, antibiotics, microbiota, germ-free, animal model, imiquimod, intestine, skin

## Abstract

Psoriatic patients have altered microbiota, both in the intestine and on the skin. It is not clear, however, whether this is a cause or consequence of the disease. In this study, using an experimental mouse model of psoriasis induced by imiquimod (IMQ), we show that oral treatment with a broad spectrum of antibiotics (MIX) or metronidazole (MET) alone mitigates the severity of skin inflammation through downregulation of Th17 immune response in conventional mice. Since some antibiotics, including MET, can influence immune system reactivity, we also evaluated the effect of MIX in the same model under germ-free (GF) conditions. GF mice treated with MET did not show milder signs of imiquimod-induced skin inflammation (IISI) which supports the conclusion that the therapeutic effect is mediated by changes in microbiota composition. Moreover, compared to controls, mice treated with MIX had a significantly higher abundance of the genus *Lactobacillus* in the intestine and on the skin. Mice treated with MET had a significantly higher abundance of the genera *Bifidobacterium* and *Enterococcus* both on the skin and in the intestine and of *Parabacteroides distasonis* in the intestine. Additionally, GF mice and mice monocolonized with either *Lactobacillus plantarum* or segmented filamentous bacteria (SFB) were more resistant to IISI than conventional mice. Interestingly, compared to GF mice, IMQ induced a higher degree of systemic Th17 activation in mice monocolonized with SFB but not with *L. plantarum*. The present findings provide evidence that intestinal and skin microbiota directly regulates IISI and emphasizes the importance of microbiota in the pathogenesis of psoriasis.

## Introduction

Psoriasis is one of the most common immune-mediated inflammatory disorders of the skin that occurs in genetically predisposed individuals (Mak et al., 2009). It affects around 2–3% of the world population, and its incidence has been recently tightly linked to metabolic syndrome and other systemic inflammatory diseases (Armstrong et al., 2013; Boehncke, 2018). Psoriasis onset is triggered mainly by environmental factors, such as stress, bacterial infection, diet, and antibiotics (e.g., tetracycline) (Tsankov et al., 1988; Zeng et al., 2017). Recently, microbes and composition of microbiota have been prominently implicated in the etiopathogenesis of this disease (Yan et al., 2017). Several studies have demonstrated the differences in skin microbiota composition between healthy individuals and psoriatic patients (both in lesions and clinically unaffected skin), indicating that psoriasis can affect skin microbiome composition all over the human body (Tett et al., 2017; Yan et al., 2017). The importance of microbiota in psoriasis induction and pathogenesis is further highlighted by the observation that microbial infections are often associated with the development and/or aggravation of psoriasis (Naldi et al., 2001; McFadden et al., 2009). Disease exacerbation can be also associated with skin or mucosa streptococcal infection and colonization with *Staphylococcus aureus*, *Malassezia*, or *Candida albicans* (Noah, 1990; Waldman et al., 2001; Weisenseel et al., 2002).

Alteration of intestinal microbiota changes the systemic proinflammatory status of the host (Tlaskalova-Hogenova et al., 2011). Based on our studies, colonization of the gastrointestinal tract of germ-free (GF) animals with one bacterial strain or complex intestinal microbiota influences the host immune system at the local and systemic level, promoting proinflammatory or anti-inflammatory response, depending on the species used (Tlaskalova-Hogenova et al., 2011). The importance of the gut-skin axis in pathogenesis of psoriasis has been recently documented in humans as well as in animal models of psoriasis (Fry et al., 2013; Zanvit et al., 2015; Vlachos et al., 2016; Zakostelska et al., 2016; Drago et al., 2018). Recovery from intestinal dysbiosis, e.g., by healing the syndrome of small intestinal bacterial overgrowth, may mitigate the symptoms of psoriatic patients (Drago et al., 2018). Outbreaks of plague psoriasis may be connected to bacterial translocation into bloodstream which may result from increased intestinal permeability in psoriatic patients (Ramírez-Boscá et al., 2015). Moreover, changes in intestinal microbial diversity found in patients with IBD and obesity, particularly reduced abundance of *Akkermansia muciniphila*, have been recently observed also in patients with psoriasis (Tan et al., 2018).

To date, no study has investigated a potential causal relationship between changes in the gut and/or skin microbiota and psoriasis development and progression. However, numerous mice and human studies provide evidence for the influence of intestinal bacteria on skin condition (Salem et al., 2018). The important role of the skin-gut axis is highlighted by the findings that mice fed with the probiotic bacterium *Lactobacillus reuteri* developed thicker skin and denser and shinier fur and regained better reproductive fitness (Levkovich et al., 2013; Erdman and Poutahidis, 2014). Our previous research showed that broad spectrum antibiotic treatment (MIX) in conventional and GF mice leads to better resistance to imiquimod (IMQ)-induced skin inflammation (IISI) (Zakostelska et al., 2016). This effect goes hand in hand with downregulation of Th17 response. Moreover, the ATB treatment dramatically changed the diversity of intestinal bacteria, with an increase in Lactobacillales and a significant decrease in Coriobacteriales and Clostridiales (Zakostelska et al., 2016). Similarly, Zanvit et al. (2015) reported that antibiotic treatment in adult but not newborn mice resulted in amelioration of IISI. Moreover, the disease in neonatally ATB-treated mice was less severe when they were co-housed with untreated controls before the IISI induction, suggesting a protective role of unperturbed microbiota (Zanvit et al., 2015).

In the present study, we aim to investigate whether the individual constituents of antibiotic mixture used in our previous work have the potential to mitigate IISI on their own and to examine the resulting changes in microbiota composition and in the immune response both on the skin and in the intestine. Furthermore, we monocolonized mice with a well-known probiotic species *Lactobacillus plantarum* WCFS1 (LP) or with segmented filamentous bacteria (SFB) and compared them with conventional and GF mice to explore how a microbial diversity impact the severity of IISI.

## Materials and Methods

### Mice

We used female BALB/c or C57BL/6 mice (7–10 weeks old) reared either in conventional or GF conditions at the Institute of Microbiology of the CAS. Mice were fed with Altromin 1,414 diet (Altromin, Lage, Germany; irradiated with 59 kGy for 30 min) and provided sterile water *ad libitum*. The GF mice were reared in sterile Trexler-type plastic isolators for several generations before being used in the experiments. Fecal samples were evaluated weekly by standard microbiological techniques to detect any contamination by bacteria, viruses, molds, and yeasts (Hrncir et al., 2008). The animals were kept in a room with a 12 h light-dark cycle at 22 °C (Kozakova et al., 2016). All experiments were approved by the Animal Care and Use Committee at the Institute of Microbiology, CAS, approval IDs: 34/2017 and 39/2015.

### Monoassociation of Germ-Free Mice

We cultured *L. plantarum* WCFS1 in MRS broth (Oxoid, Basingstoke, UK) overnight. Then, we centrifuged the culture and washed it in sterile phosphate-buffered saline (PBS). We adjusted the concentration to 10^9^ CFU/ml. After weaning, the BALB/c GF mice were colonized intragastrically by 2 × 10^8^ CFU/0.2 ml of lactobacilli suspension. The colonization level of the animals was checked regularly by culturing their feces: appropriate serial dilutions were plated on MRS agar plates and colonies were counted after incubation at 37 °C for 48 h. Colonization remained stable throughout the whole experiment and reached levels of 2–3 × 10^9^ CFU/g feces. The littermates (second or third generation) of monocolonized mice were used for the experiments (Schwarzer et al., 2019). Monocolonization with SFB was described previously (Stepankova et al., 2007). Briefly, after weaning, C57BL/6 GF mice were colonized intragastrically with viable SFB (10^7^–10^8^ per dose) obtained from the stool of mice monoassociated with SFB. To check for the presence of SFB in the colon and cecum, we used an *in situ* hybridization probe SFB 1008-FITC (sequences 5′-GCGAGCTTCCCTCATTACAAGG-3′) (Snel et al., 1995).

### Mouse Model of Psoriasis

To induce skin inflammation, the mice were treated daily for up to six to seven consecutive days with 62.5 mg of IMQ cream (Aldara, 3M Health Care Limited, Great Britain), applied on the shaved back skin and left ear. The severity of erythema and scaling was monitored daily, using a scale based on the clinical Psoriasis Area and Severity Index (PASI). Ear swelling and skin thickening were measured at the end of the experiment, as previously described (van der Fits et al., 2009). Histopathological examinations were performed in 4 μm sections stained with hematoxylin/eosin by an experienced pathologist (PR), who was blinded to the treatment status of the mice. The scoring system describing the degree of imiquimod-induced skin inflammation (IISI) on a scale of 0–2 has been previously developed in our laboratory (Zakostelska et al., 2016).

### Antibiotic Treatment

**Figure 1:**
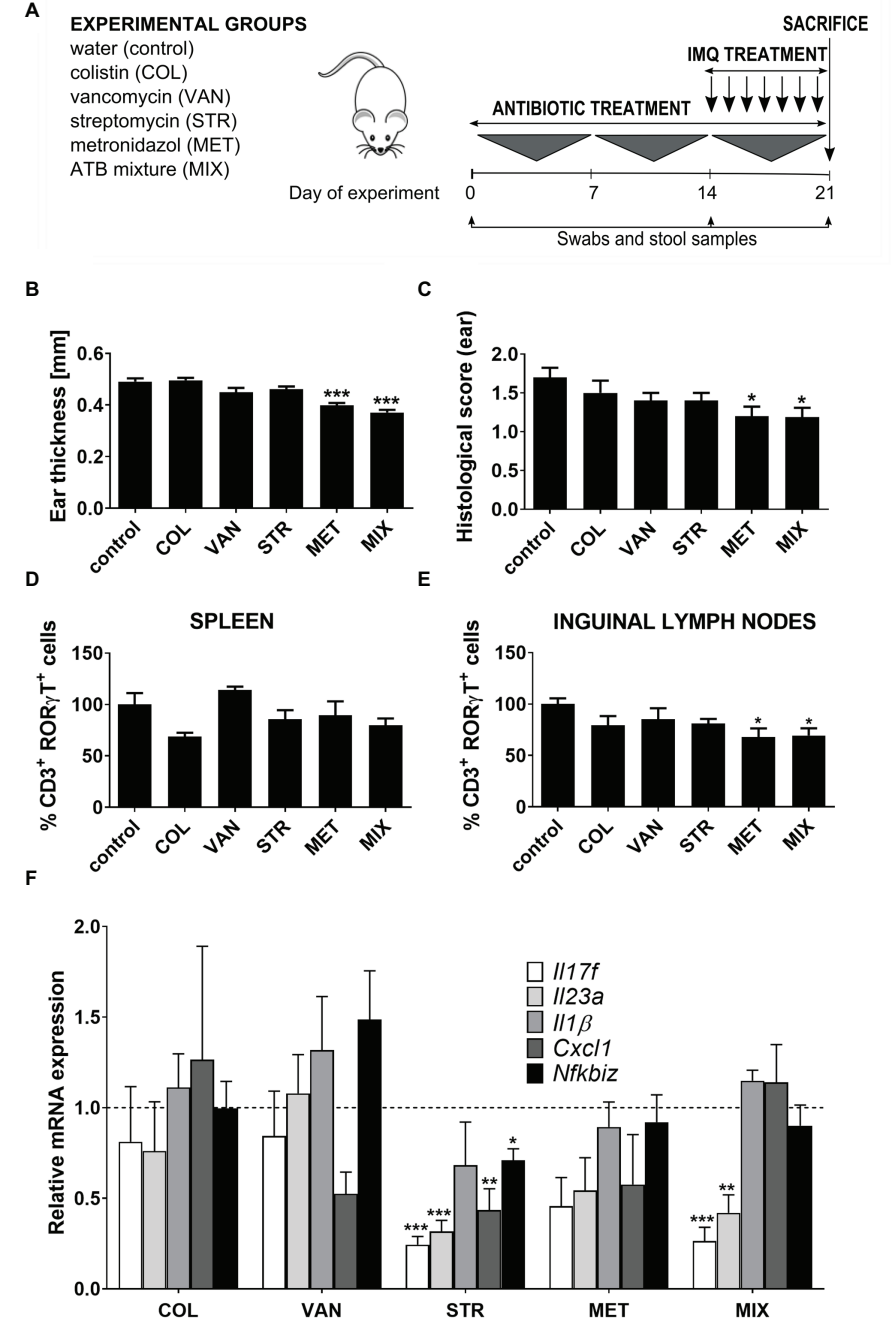
Treatment with a single antibiotic or antibiotic mixture changes the susceptibility to IMQ-induced skin inflammation in BALB/c mice. **(A)** Experimental design: Female BALB/c mice were divided into six groups (five mice per group) and treated perorally with colistin (COL), vancomycin (VAN), streptomycin (STR), metronidazole (MET), or MIX of these antibiotics for 21 days. Starting on Day 14, mice were treated with IMQ until the end of experiment. **(B,C)** Quantification of ear thickness and histopathological score at the end of experiment (Day 21). **(D,E)** Flow cytometric analysis of expression of CD3^+^RORγt^+^ T cells in spleen and inguinal nodes. Percentage is relative to the proportion of live cells gated on CD3^+^ and subsequently on RORγt^+^ in control mice.100% represents controls gated on live CD3^+^RORγt^+^. **(F)** Quantitative PCR analysis of mRNA expression of *Il17f, Il23a, Il1b, Cxcl1,* and *Nfkbiz* in the skin. Data were normalized to the expression of the Elongation factor 2 (*Eef2*) as a reference gene. Five mice per group were analyzed, and representative data from one out of three independent experiments are shown. Statistical differences between the groups were determined by ANOVA **(B–E)** or by Student’s *t*-test **(F)**. *p < 0.05, **p < 0.01, and ***p < 0.001.

Mice were treated daily with antimicrobials, starting 2 weeks prior to psoriasis induction until the end of the experiment (see experimental design in Figure 1A) (Zakostelska et al., 2016). Mice in each of the five experimental groups were given 300 μl of either metronidazole (5 mg/ml; B. Braun, Melsungen AG, Germany), vancomycin (5 mg/ml), colistin (1.66 mg/ml, Sigma-Aldrich), streptomycin (50 mg/ml, Sigma-Aldrich), or a mix of these antimicrobials by oral gavage. Administration by gavage was chosen in order to prevent severe dehydration and weights loss caused by unwillingness to drink water containing antimicrobials and to prevent the mice from bathing in the antibiotics. This treatment was well tolerated by all mice (Reikvam et al., 2011).

### Microbiota Analysis

Changes in microbiota composition were analyzed in swabs from psoriatic-like skin lesions and in stool samples of experimental mice. Briefly, samples were collected from all mice at the beginning of the experiment (Day 0), after 14 days of ATB treatment before the IMQ application (Day 14), and after 7 days of IMQ application (Day 21). We isolated DNA from both swabs (PowerBiofilm DNA Isolation Kit, MoBio) and stool samples using the MasterPure™ Complete DNA and RNA Purification Kit (Epicentre, Madison, WI, USA). Next, we amplified the V3-V4 region of 16S rRNA gene using degenerate primers 341F (5′-CCTACGGGNGGCWGCAG-3′) and 806R (5′-GGACTACHVGGGTWTCTAAT-3′), which were barcoded to enable multiplexing of sequencing libraries. Subsequently, we processed them by PCR amplification, plate purification of amplicons, and ligating of adapters as previously described (Zakostelska et al., 2016). We quantified the amplicon library using the KAPA Library Quantification Kit (Illumina) and sequenced on MiSeq platform using 2 × 300 bp kit at the CEITEC Genomics Core Facility (Brno, CZ). Sequencing data were processed using QIIME (Quantitative Insights Into Microbial Ecology) software package version 1.9.1 (Caporaso et al., 2010). The data are available in the Sequence Read Archive (SRA), http://www.ncbi.nlm.nih.gov/sra, under the accession number SRP156846. For microbiota analysis, Shannon index and weighted and unweighted UniFrac distances expressed in the form of PCoA plots were used to describe alpha and beta diversity. To determine the discriminative features for both taxonomic profiles of communities, the LEfSe analysis tool was employed (Caporaso et al., 2010).

### Flow Cytometry Analysis of the Induced Immune Response

Single cell suspensions of the spleen and inguinal lymph nodes were prepared and blocked as previously described (Zakostelska et al., 2011). The cells were then stained extracellularly with FITC-conjugated anti-CD3 (clone 145-2C11, dilution 1:100), and dead cells were excluded using the fixable viability dye eFluor 780 (dilution 1:200). Subsequently, the cells were fixed, permeabilized, and stained intracellularly with PE-conjugated anti-RORγt (clone AFJKS-9, dilution 1:50). Flow cytometry analysis was performed using LSRII (BD Bioscience) and evaluated by FlowJo software v 9.6.2. (Tree Star, Inc., Ashland, OR).

### Gene Expression in the Skin/RNA Isolation and Quantitative Real-Time PCR

Total RNA was isolated from approx. 50 mg of mouse skin tissue using the RNeasy Mini Kit (Qiagen) according to the manufacturer’s protocol. RNA samples were treated with TURBO DNA-free Kit (Thermo Fisher Scientific), and 400 ng of total RNA was reverse transcribed using oligo(dT)20 primers and SuperScript IV Reverse Transcriptase (Thermo Fisher Scientific). The resulting cDNA served as a template for qPCR analysis with the CFX96 Real-Time PCR detection system (BioRad) using the iQ SYBR Green Supermix (BioRad). Each PCR reaction was performed in duplicates in a volume of 25 μl containing 4 μl of a 1:10 dilution of each cDNA preparation, 12.5 μl of SYBR Green Supermix, and 0.2 μM of each primer. The amplification protocol was as follows: 3 min at 95 °C followed by 40 cycles at 94 °C for 30 s, 60 °C for 35 s, and 72 °C for 50 s. The temperature was then gradually increased to 95 °C to obtain melting curves of the amplified fragments, which confirmed the specificity and uniformity of the PCR products. Serial dilutions of cDNA (1:5) were used in qPCR for each primer pair to determine the efficiency of amplification. Changes in gene expression were calculated using the 2^−ΔΔCT^ (Livak) method. Quantitative measurements were normalized using elongation factor 2 (*Eef2*) mRNA levels as the reference gene. Changes in mRNA levels were shown as the fold change of expression in monocolonized mice compared to that in conventional mice. Data were expressed as mean ± SEM of the values obtained in all experiments.

### Statistical Analysis

We used unpaired Student’s *t*-test to compare two experimental groups or one-way analysis of variance (ANOVA) with Dunnett’s multiple comparison test to compare multiple groups.

All data are expressed as the mean ± standard deviation (SD) unless otherwise stated, and differences were considered statistically significant at *p* ≤ 0.05. For analyses, we used the GraphPad Prism statistical software (version 5.0, GraphPad Software, Inc., La Jolla, CA, USA).

## Results

### MET Decreases the Sensitivity to IISI Similarly as MIX

We have previously shown that the severity of IISI can be decreased by oral treatment with a broad-spectrum antibiotics mixture (Zakostelska et al., 2016), and here, we investigate the contribution of each of its components to the overall effect. We treated the animals with placebo (saline), or either metronidazole (MET), vancomycin (VAN), colistin (COL), streptomycin (STR), or their mixture (MIX) for 21 days. During the last 6 days of this treatment, we induced skin inflammation by daily application of IMQ on their ear and shaved back skin (Figure 1A). Similarly to the MIX-treated mice, mice treated with MET had significantly milder skin inflammation compared to controls (Figures 1B,C). There was some, albeit non-significant, decrease in disease severity in STR- and VAN-treated mice as well, which suggests a synergic effect responsible for the slightly stronger and more robust effect of MIX. On the other hand, COL-treated mice had psoriasis-like symptoms of comparable severity as control mice, including parakeratosis, acanthosis with prominent gothic vaults, or focal microabscesses. On the contrary, mice treated with MIX or MET exhibited only low-degree skin thickening, mild hyperkeratosis, and acanthosis of epidermis, but without parakeratosis and accumulation of leukocytes in corium, as shown by histological examination (Supplementary Figure 2) and quantification summarized in Figure 1C. Since Th17 cells play a crucial role in this model, we analyzed the impact of these antibiotics on the proportion of CD3^+^RORγt^+^ cells in spleen and inguinal lymph nodes by flow cytometry (for gating strategy, see Supplementary Figure 1). While there were no significant changes in the systemic circulation (spleen), MET decreased the proportion of CD3^+^RORγt^+^ cells to a similar degree as MIX (Figures 1D,E). Again, neither COL nor VAN decreased the proportion of CD3^+^RORγt^+^ cells as compared to controls. To further investigate these data, we analyzed the expression of key proinflammatory factors (*Il17f, Il17a, Il23a, Il1b, Cxcl1, Rorγt,* and *Nfkbiz*) directly in the skin by RT-qPCR. We found that MIX and STR reduced the expression of *Il23a* and *Il17f*, while neither VAN nor COL had this effect (Figure 1F). There was a trend toward reduced expression of *Il23a* and *Il17f* or *Il17a* in MET-treated group (Figure 1F and Supplementary Figure 3A). Nevertheless, none of the treatments reduced the expression of transcription factor *Rorγt* (Supplementary Figure 3B). There may be some differences in the immunomodulatory effects of these antibiotics, since STR treatment seemed to lead to a generalized decrease in the expression of proinflammatory factors, which was significantly lower for all genes but not for *Il1b*. All these results together suggest that most of the effect of MIX on the IISI is conveyed by MET, and the presence of VAN, COL, and (to some extent) STR is largely inconsequential.

### Microbiota Is Necessary for the Protective Effect of MET in IISI

**Figure 2:**
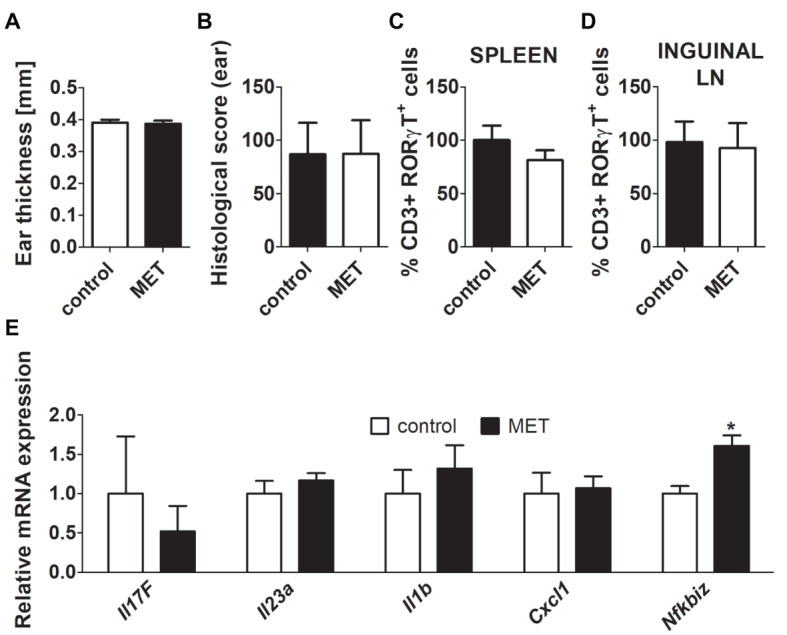
Treatment by metronidazole did not change the severity of inflammation after IMQ application in germ-free (GF) BALB/c mice. **(A,B)** Quantification of ear thickness and ear histological score in GF controls and GF mice treated by metronidazole. **(C,D)** Comparison of the expression of CD3^+^RORγT^+^ in GF controls versus GF mice treated by metronidazole in spleen or inguinal lymph nodes. Percentage is relative to the proportion of live cells gated on CD3^+^ and subsequently on RORγt^+^ in control mice. 100% represents controls gated on live CD3^+^RORγt^+^. **(E)** Quantitative PCR analysis of mRNA expression of *Il17f, Il23a, Il1b, Cxcl1,* and *Nfkbiz* in the skin. The graphs show the results of one representative experiment out of two independent experiments (*n* = 6–7 mice per group). Differences between MET-treated group and control group were determined by unpaired Student’s *t*-test **(A–E)**. *p < 0.05.

Oral antibiotics may possess immunomodulatory properties when administered in high concentrations (Grove et al., 1977; Al-Banna et al., 2013). Therefore, we analyzed whether MET influences the IISI in a microbiota dependent or independent manner, by repeating the experiments under GF conditions. There were no differences in disease severity or Th17 proportions between MET-treated and control GF mice (Figures 2A–D). This suggests that it is the antimicrobial activity of MET that is responsible for its anti-inflammatory effect. Interestingly, there was only one statistically significant difference between these two groups, *Nfkbiz* expression in the skin is higher in MET-treated GF mice than in controls, suggesting that there is some minor, immunomodulatory microbiota-independent effect of the drug (Figure 2E).

### Treatment With Some Oral Antibiotics Leads to Profound Changes in the Intestine, but Not in the Skin Microbiome

**Figure 3:**
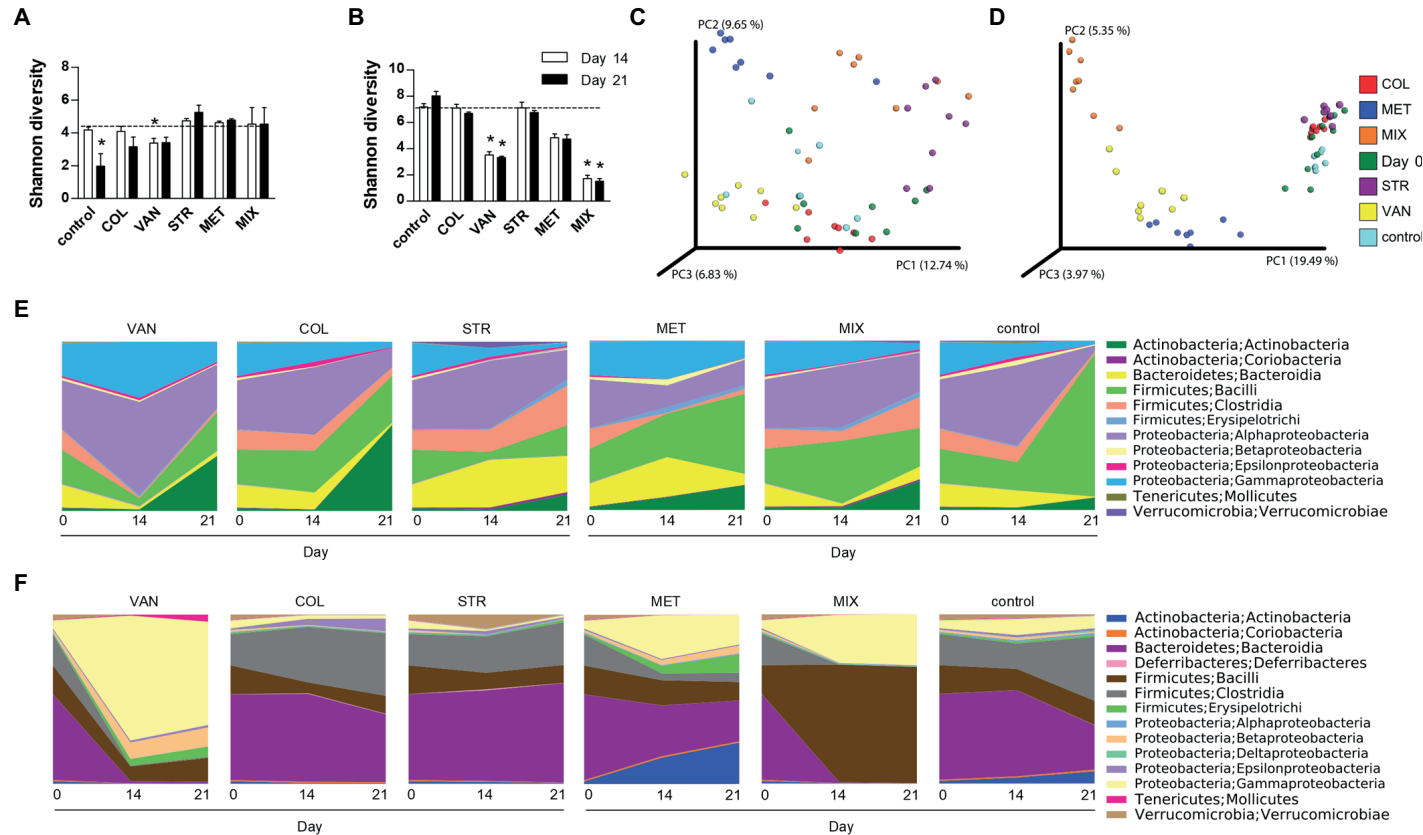
Antibiotic treatment caused extensive shifts in microbial communities in the intestine but not on the skin. Differences in bacterial diversity in the skin **(A)** and intestine **(B)** among the groups treated with antibiotics and the control group of mice expressed by Shannon diversity index. Dashed line is background of bacterial diversity at Day 0. Data represent means ± SD from the pool of either 4–5 mice (Day 14) or 3 mice (Day 21) in each group. Statistical significance was determined by unpaired Student’s *t*-test; *p < 0.05 and **p < 0.01. Principal coordinates analysis (PCoA) plot using the unweighted UniFrac distance metric compares the compositional similarity between all tested groups in the skin **(C)** and intestine **(D)**. Groups are color-coded and each point represents one mouse. Statistical significance was confirmed using PERMANOVA. Time-dependent changes in microbial composition on the skin **(E)** or in the intestine **(F)**. Relative bacterial abundances are shown as a mean of group of either 7–10 mice (Day 0), 4–5 mice (Day 14), or 3 mice (Day 21). COL, colistin; MET, metronidazole; VAN, vancomycin; STR, streptomycin; MIX, antibiotic mixture. Day 0 shows the composition of mouse microbiota at the beginning of the experiment, i.e. before antibiotics treatment (Day 0); Day 14 follows microbiota composition after two weeks of each antibiotic treatment (Day 14); and Day 21 shows microbiota after the induction of skin inflammation (Day 21).

We analyzed microbiota composition on the skin and in the intestine before the start of the ATB treatment (Day 0), before the IMQ application (Day 14), and at the end of the experiment (Day 21). Skin swabs and fecal samples were analyzed by sequencing the V3-V4 regions of the 16S rRNA gene. The mice treated with VAN and MIX reported significant reduction of microbial richness and evenness in the intestine but not on the skin (Figures 3A,B). There was similar trend toward decreased microbial richness and evenness in the intestine in MET-treated group (Figure 3B). Microbial diversity on the skin was significantly changed only after IMQ treatment in control or VAN group at the end of the experiment compared to microbiota composition on Day 0 (Figure 3A). The compositional similarity revealed that the skin (Figure 3C) and intestinal (Figure 3D) microbiota profiles after MET or MIX treatment, regardless of the presence or absence of the disease (Day 14 or Day 21), were strikingly different (*p* = 0.001) from those of the other tested groups and from the microbiota composition at the beginning of experiment. Consistently with our previous results (Zakostelska et al., 2016), antibiotic treatment did not decrease the total amount of bacteria (Supplementary Figure 4A). This suggests that both microbial composition and microbial load play important roles in psoriasis improvement. Antibiotic treatment and IMQ application generally changed the composition of microbiota in the intestine and on the skin (Figures 3E,F). MIX reduced the diversity of microbiota composition, and the vacant niche in the intestine was filled by Firmicutes, especially Lactobacillales, even though Clostridiales and Bacillales declined. Additionally, treatment with MIX led to reduction of Coriobacteriales in the intestine, in line with our previous results (Zakostelska et al., 2016). Similarly to the MIX-treated group, mice in the MET-treated group showed lower abundance of the family Ruminococcaceae, Clostridiales, and genus *Oscillospira* and *Dorea* in the intestine compared to the control group (Figures 3E,F). LefSe analysis revealed several discriminative features between MIX, MET, and control groups. For selected representatives, see Table 1; for detailed analysis, see Supplementary Tables 1 and 2. MET, but not MIX, treatment significantly increased the presence of *Parabacteroides distasonis* in the intestine. Moreover, MET significantly increased the abundance of the genera *Bifidobacterium* and *Enterococcus* compared to MIX or control groups, both in the intestine and on the skin (Table 1). Mice in the MIX group had a significantly higher abundance of genus *Lactobacillus* in the intestine and on the skin compared to controls (Table 1). Since significant reduction of *A. muciniphila* has been described in the intestine of patients with psoriasis (Tan et al., 2018), we analyzed the relative abundance of this species in our samples. We found that IMQ treatment itself did not significantly change the abundance of *A. muciniphila,* but treatment with VAN or MIX led to its eradication (Supplementary Figure 5). These results suggest that specific bacterial composition affects the severity of IMQ-induced inflammation.

**Table 1:** Selective distinctive features of changes in skin or intestinal microbiota composition distinguished by LEfSe (g_genus, s_species).

Distinctive composition of mouse microbiota using LEfSe				
	Day	Control	MIX	MET
Skin	14	–	g_*Lactobacillus*	g_*Enterococcus* g_*Bifidobacterium*
	21	–	g_*Lactobacillus*	g_*Enterococcus* g_*Bifidobacterium*
Feces	14	–	g_*Lactobacillus*	g_*Bifidobacterium* g_*Enterococcus* s_*Parabacteroides distasonis*
	21	–	g_*Lactobacillus*	g_*Bifidobacterium* s_*Parabacteroides distasonis* g_*Enterococcus*

### Broad Spectrum of Bacterial Antigens and Fully Matured Immune System Is Required for IISI Development

**Figure 4:**
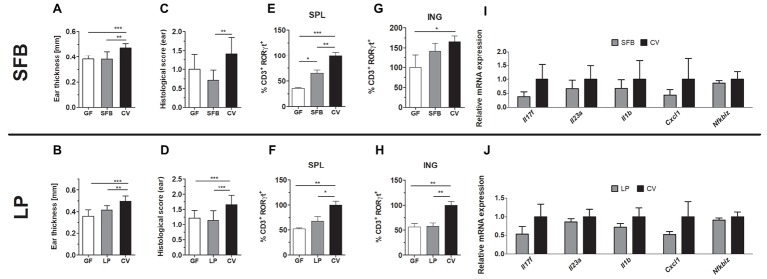
Mice monocolonized with SFB and germ-free (GF) C57BL/6 or mice monocolonized with *Lactobacillus plantarum* (LP) and germ-free (GF) BALB/c mice show decreased imiquimod-induced skin inflammation (IISI) in comparison to conventional mice (CV). **(A,B)** Quantification of ear thickness. **(C,D)** Quantification of histopathological score after H&E staining of the ear. Comparison of the expression of CD3^+^RORγT^+^ in GF and SFB or LP versus CV mice in the spleen **(E,F)** or inguinal lymph nodes **(G,H)**. Percentage is relative to the proportion of live cells gated on CD3^+^ and subsequently on RORγt^+^ in control mice.100% represents controls gated on live CD3^+^RORγt^+^. **(I,J)** Quantitative PCR analysis of mRNA expression of *Il17f, Il23a, Il1b, Cxcl1,* and *Nfkbiz* in the skin. The graphs show a representative experiment (*n* = 6–7 mice per group). Statistical differences between the groups were determined by ANOVA **(A–H)** or Student’s *t*-test **(I,J)**; *p < 0.05, **p < 0.01, and ***p < 0.001.

We found that genus *Lactobacillus* is significantly increased in the intestine following the protective MIX treatment (Supplementary Figure 4B). Since *L. plantarum* has been previously used in the treatment of inflammatory skin diseases, here we further investigated whether monocolonization with *L. plantarum* WCFS1 (LP) itself changes the course of IISI compared to GF and conventional mice (Jang et al., 2014; Mariman et al., 2016). We found that monocolonization with LP led to a similar degree of skin inflammation as in GF mice in all the tested parameters. All these parameters were also significantly lower than in conventional mice (Figures 4B,D,F,H) or there was a trend toward reduced relative mRNA expression of *Il17f, Il23a, Il1b, Cxcl1,* and *Nfkbiz* (Figure 4J). Additionally, inflammation or VAN, STR, and MIX decreased the abundance of SFB in the intestine of conventional mice (Supplementary Figure 4C). Since MIX showed the greatest efficiency in mitigating the inflammation and SFB are known for their Th17 inducing capacity, we monocolonized mice with SFB to investigate their role in IISI. SFB and GF mice did not differ in clinical signs on the skin, except the CD3^+^RORγT^+^ cell proportion, which was higher in the spleen of SFB mice (Figures 4A,C,E,G,I). As compared to conventional mice, SFB-monocolonized mice had milder inflammation in all tested parametres or there was a trend toward decreased expression of CD3^+^RORγT^+^ in inguinal lymph nodes (Figure 4G) or reduced relative mRNA expression of *Il17f, Il23a, Il1b, Cxcl1,* and *Nfkbiz* (Figure 4I). These results indicate that monocolonization with a single bacterial species or with a small group of specific bacteria, even though they possess some proinflammatory potential, may not be enough to induce full signs of IISI as observed in conventional mice.

## Discussion

Microbiota fundamentally influences the immune system development, and its perturbation, i.e., dysbiosis, is associated with many inflammatory diseases (Tlaskalova-Hogenova et al., 2011). While it is well established that the skin microbiota is involved in the pathogenesis of psoriasis (Fry et al., 2013), much less is known about the effect of intestinal microbiota on skin inflammation. By comparing germ-free and conventional mice, we have previously shown that the presence of intestinal microbiota promotes the IISI in mice by enhancing Th17 response and that by oral administration of broad-spectrum antibiotic mixture, a similar effect as in germ-free mice can be achieved (Zakostelska et al., 2016). Here, we analyzed which antibiotic drives this beneficial effect, which microbes are affected by this treatment, and whether the effect is microbiota-dependent or independent.

First, we found that MET has similar beneficial effect on IISI as the whole mixture of antibiotics. Since no other antibiotic contained in the MIX was able to reduce the severity of IISI on its own, we conclude that most of the protective effect of the MIX is due to MET. Both successful treatments – oral MET and MIX – decreased the proportion of Th17 cells (CD3^+^RoRγT^+^) in the draining lymph nodes. Moreover, treatment with STR, MET, or MIX changed the expression of proinflammatory cytokines in the inflamed skin. Similarly to MIX, STR decreased cutaneous expression of *Il17f* and *Il23a*, but, unlike MIX or MET, it failed to improve IISI severity. MET slightly decreased *Il17f* and *Il23a* expression as well, but this effect was not statistically significant. This suggest that cutaneous Th17 response is not the only factor behind the beneficial effect of antibiotics.

Although microbes markedly change the reactivity of the immune system, MET may affect inflammation by its anti-inflammatory action (Al-Banna et al., 2013; Becker et al., 2016). MET is used as a supplemental treatment to patients with Crohn’s disease, where it decreases the severity of early disease recurrence after ileum resection and improves healing of perianal fistulae (Rutgeerts et al., 1995; Dejaco et al., 2003). Nevertheless, MET is not efficient as a Crohn’s disease monotherapy (i.e., without concurrent anti-inflammatory treatment), and even if combined with ciprofloxacin, its effect is only limited to a less common colonic Crohn’s disease (Steinhart et al., 2002). The well-established involvement of gut microbiota in Crohn’s disease pathogenesis and efficiency of oral MET in the treatment of small bowel bacterial overgrowth (common complication of Crohn’s disease) suggest that MET’s anticolitic effect is driven by its antimicrobial and not anti-inflammatory action (Castiglione et al., 2003). The effect of oral antibiotics on the inflammation outside gut may be less obvious, because mechanisms such as T cell polarization may be dependent on microbiota (Furusawa et al., 2013; Smith et al., 2013; Viaud et al., 2013; Scott et al., 2018), other mechanisms may not. In our previous studies, we found that oral treatment of mice with mixture of antibiotics, including MET, decreases severity of skin or retinal inflammation similarly as GF state (Heissigerova et al., 2016; Zakostelska et al., 2016). In humans, oral MET was successfully used as treatment for idiopathic lichen planus, even without clear parasitic infection (Wahba-Yahav, 1995; Buyuk and Kavala, 2000; Rasi et al., 2010). These results indicate that MET may have immunomodulatory effect in skin inflammatory disease beyond its antiparasitic effect, but none of these studies controlled for its ability to kill bacteria, mainly anaerobes. While skin and respiratory infections may play a role in triggering psoriatic disease, exposure to MET is not independently associated with disease risk (Horton et al., 2016). It is still unclear, if MET could influence the IISI directly, by immunomodulation or indirectly, by antibiotic effect. Therefore, we induced IISI in mice treated with MET under GF conditions and compared its severity with mice treated with placebo. Although the IISI is significantly less severe in GF than in conventional mice, oral MET did not have any effect on the severity of the inflammation in any of the studied parameters, suggesting that the anti-inflammatory effect of MET is microbiota dependent.

Next, we analyzed the changes in both intestinal and skin microbiome induced by antibiotics or IISI to identify the key microbes involved. Oral antibiotics induced significant changes in gut microbiota, but only minor changes in skin microbiota, which decreased in diversity only with VAN treatment. Mice may actively transfer the oral antibiotics directly on their skin by bathing in the drinking water or by licking their backs (O’Neill et al., 2016). We administered the antibiotics by gavage, thereby avoiding this issue. The absence of similar decrease in other groups implies that this decrease in microbial diversity is caused by a different mechanism. There is a clear decrease in skin microbial diversity during the IISI development in the control group, which is probably the consequence of severe skin inflammation in these mice. Similar tendency was found in patients with inflammatory skin diseases such as psoriasis and atopic dermatitis (Kong et al., 2012; Alekseyenko et al., 2013). Similar to other studies in humans, we found that milder skin inflammation in mice is associated with higher abundance of Proteobacteria and lower abundance of Staphylococci and Streptococci in the MET- and MIX-treated groups (Gao et al., 2008; Alekseyenko et al., 2013). Reduction of *A. muciniphila* in the intestine has been recently found in patients with psoriasis (Tan et al., 2018). It indicates an interesting link between skin inflammation and intestinal microbiota, therefore we analyzed its changes during IISI induction. However, in contrast to the human studies, we did not find any significant changes in *A. muciniphila* during IISI induction, proposing differences between the mouse model of psoriasis and human disease.

In the intestine, both VAN and MIX significantly decreased microbial diversity and VAN-, MET-, and MIX-induced profound shifts in β-diversity. This manifested as marked overrepresentation of lactobacilli in the intestine of both MET- and MIX-treated animals on Day 14. This is in agreement with other studies analyzing intestinal and skin microbiota in mice treated with broad-spectrum antibiotic mixtures (Zanvit et al., 2015; Zakostelska et al., 2016). Interestingly, there are anti-inflammatory probiotics among lactobacilli, which can induce regulatory T cells even in the form of lysates (Zakostelska et al., 2011). Moreover, it is frequently shown that oral treatment with *L. plantarum* has a protective effect in mouse models of skin and gut inflammation (Jang et al., 2014; Kim et al., 2015; Mariman et al., 2016).

Based on these observations, we decided to monocolonize mice either with *L. plantarum* WCFS1 (LP) or with SFB. Both are well-characterized intestinal commensals, chosen for their ability to modulate the immune system. While the former is able to regulate inflammation by inducing high levels of regulatory cytokine IL-10, the latter shifts the T cell response towards Th17 (Ivanov et al., 2009; Gorska et al., 2014). Therefore, we expected that LP would decrease the severity of IISI by IL-10-dependent immune system regulation and that SFB would aggravate IISI by stimulating the Th17 cells. Although monocolonization with SFB led to a significant increase in RORγt^+^ T cells in the mouse spleen, neither line of monocolonized mice differed from GF mice in IISI severity. The failure of SFB monocolonization to induce substantial aggravation of the IISI may be due to a similar mechanism as in experimental colitis, where SFB can worsen colitis only in the presence of other commensals (Stepankova et al., 2007). Taken together, these results indicate that microbial diversity is crucial for a full-fledged immune response in IISI.

## Conclusion

In summary, we suggest that MET and MIX are sufficient to decrease the severity of IISI in a microbiota-dependent manner. While these beneficial changes are accompanied with downregulation of Th17 activity and an increase in abundance of intestinal lactobacilli in group treated with antibiotic MIX, monocolonization with neither lactobacillus nor Th17-promoting SFB is sufficient to change the IISI severity. These results emphasize the importance of gut-skin axis in the pathogenesis of inflammatory skin diseases. Our future studies will focus on causative effects of the changes in microbiota and more detailed study of antigen recognition and immune response to modulators released by bacteria mediating these changes. These data suggest a therapeutic potential *via* influencing the microbiota composition in psoriatic patients.

## Author Contributions

HT-H, JH, and ZZ conceived and designed the research. ZS, KK, PR, JD, SC, DS, PP, TH, HK, RS, FR, KJ, and ZZ performed the experiments. ZS, IN, MKo, MKv, JD, and ZZ analyzed and interpreted the data. ZS, KK, MKv, and ZZ wrote the manuscript. All authors revised and approved the final version of the manuscript.

### Conflict of Interest Statement

The authors declare that the research was conducted in the absence of any commercial or financial relationships that could be construed as a potential conflict of interest.

## References

[ref1] Al-Banna N. A. Pavlovic D. Grundling M. Zhou J. Kelly M. Whynot S.. (2013). Impact of antibiotics on the microcirculation in local and systemic inflammation. Clin. Hemorheol. Microcirc. 53, 155–169. 10.3233/CH-2012-1583, PMID: 22975936

[ref2] Alekseyenko A. V. Perez-Perez G. I. De Souza A. Strober B. Gao Z. Bihan M. (2013). Community differentiation of the cutaneous microbiota in psoriasis. Microbiome 1:31. 10.1186/2049-2618-1-31 24451201 PMC4177411

[ref3] Armstrong A. W. Harskamp C. T. Armstrong E. J. (2013). Psoriasis and metabolic syndrome: a systematic review and meta-analysis of observational studies. J. Am. Acad. Dermatol. 68, 654–662. 10.1016/j.jaad.2012.08.015 23360868

[ref4] Becker E. Bengs S. Aluri S. Opitz L. Atrott K. Stanzel C. (2016). Doxycycline, metronidazole and isotretinoin: do they modify microRNA/mRNA expression profiles and function in murine T-cells? Sci. Rep. 6:37082. 10.1038/srep37082 27853192 PMC5113073

[ref5] Boehncke W. H. (2018). Systemic inflammation and cardiovascular comorbidity in psoriasis patients: causes and consequences. Front. Immunol. 9:579. 10.3389/fimmu.2018.00579 29675020 PMC5895645

[ref6] Buyuk A. Y. Kavala M. (2000). Oral metronidazole treatment of lichen planus. J. Am. Acad. Dermatol. 43, 260–262. 10.1067/mjd.2000.104683 10906648

[ref7] Caporaso J. G. Kuczynski J. Stombaugh J. Bittinger K. Bushman F. D. Costello E. K. (2010). QIIME allows analysis of high-throughput community sequencing data. Nat. Methods 7, 335–336. 10.1038/nmeth.f.303 20383131 PMC3156573

[ref8] Castiglione F. Rispo A. Di Girolamo E. Cozzolino A. Manguso F. Grassia R. (2003). Antibiotic treatment of small bowel bacterial overgrowth in patients with Crohn’s disease. Aliment. Pharmacol. Ther. 18, 1107–1112. 10.1046/j.1365-2036.2003.01800.x 14653830

[ref9] Dejaco C. Harrer M. Waldhoer T. Miehsler W. Vogelsang H. Reinisch W. (2003). Antibiotics and azathioprine for the treatment of perianal fistulas in Crohn’s disease. Aliment. Pharmacol. Ther. 18, 1113–1120. 10.1046/j.1365-2036.2003.01793.x, PMID: 14653831

[ref10] Drago F. Ciccarese G. Indemini E. Savarino V. Parodi A. (2018). Psoriasis and small intestine bacterial overgrowth. Int. J. Dermatol. 57, 112–113. 10.1111/ijd.13797, PMID: 29057460

[ref101] Erdman S. E. Poutahidis T. (2014). Probiotic ‘glow of health’: it’s more than skin deep. Benef. Microbes 5, 109–119. 10.3920/BM2013.0042 24675231 PMC4354898

[ref11] Fry L. Baker B. S. Powles A. V. Fahlen A. Engstrand L. (2013). Is chronic plaque psoriasis triggered by microbiota in the skin? Br. J. Dermatol. 169, 47–52. 10.1111/bjd.12322, PMID: 23521130

[ref12] Furusawa Y. Obata Y. Fukuda S. Endo T. A. Nakato G. Takahashi D. (2013). Commensal microbe-derived butyrate induces the differentiation of colonic regulatory T cells. Nature 504, 446–450. 10.1038/nature12721 24226770

[ref13] Gao Z. Tseng C. H. Strober B. E. Pei Z. Blaser M. J. (2008). Substantial alterations of the cutaneous bacterial biota in psoriatic lesions. PLoS One 3:e2719. 10.1371/journal.pone.0002719, PMID: 18648509 PMC2447873

[ref14] Gorska S. Schwarzer M. Jachymek W. Srutkova D. Brzozowska E. Kozakova H. (2014). Distinct immunomodulation of bone marrow-derived dendritic cell responses to *Lactobacillus plantarum* WCFS1 by two different polysaccharides isolated from *Lactobacillus rhamnosus* LOCK 0900. Appl. Environ. Microbiol. 80, 6506–6516. 10.1128/AEM.02104-14 25107979 PMC4178633

[ref15] Grove D. I. Mahmound A. A. Warren K. S. (1977). Suppression of cell-mediated immunity by metronidazole. Int. Arch. Allergy Appl. Immunol. 54, 422–427. 328408 10.1159/000231857

[ref16] Heissigerova J. Seidler Stangova P. Klimova A. Svozilkova P. Hrncir T. Stepankova R.. (2016). The microbiota determines susceptibility to experimental autoimmune uveoretinitis. J. Immunol. Res. 2016:5065703. 10.1155/2016/5065703, PMID: 27294159 PMC4886056

[ref17] Horton D. B. Scott F. I. Haynes K. Putt M. E. Rose C. D. Lewis J. D.. (2016). Antibiotic exposure, infection, and the development of pediatric psoriasis: a nested case-control study. JAMA Dermatol. 152, 191–199. 10.1001/jamadermatol.2015.3650, PMID: 26560335 PMC4749454

[ref18] Hrncir T. Stepankova R. Kozakova H. Hudcovic T. Tlaskalova-Hogenova H. (2008). Gut microbiota and lipopolysaccharide content of the diet influence development of regulatory T cells: studies in germ-free mice. BMC Immunol. 9:65. 10.1186/1471-2172-9-65, PMID: 18990206 PMC2588440

[ref19] Ivanov I. I. Atarashi K. Manel N. Brodie E. L. Shima T. Karaoz U. (2009). Induction of intestinal Th17 cells by segmented filamentous bacteria. Cell 139, 485–498. 10.1016/j.cell.2009.09.033 19836068 PMC2796826

[ref20] Jang S. E. Han M. J. Kim S. Y. Kim D. H. (2014). *Lactobacillus plantarum* CLP-0611 ameliorates colitis in mice by polarizing M1 to M2-like macrophages. Int. Immunopharmacol. 21, 186–192. 10.1016/j.intimp.2014.04.021, PMID: 24815859

[ref21] Kim H. Kim H. R. Kim N. R. Jeong B. J. Lee J. S. Jang S.. (2015). Oral administration of *Lactobacillus plantarum* lysates attenuates the development of atopic dermatitis lesions in mouse models. J. Microbiol. 53, 47–52. 10.1007/s12275-015-4483-z, PMID: 25471185

[ref22] Kong H. H. Oh J. Deming C. Conlan S. Grice E. A. Beatson M. A.. (2012). Temporal shifts in the skin microbiome associated with disease flares and treatment in children with atopic dermatitis. Genome Res. 22, 850–859. 10.1101/gr.131029.111, PMID: 22310478 PMC3337431

[ref2300] Kozakova H. Schwarzer M. Tuckova L. Srutkova D. Czarnowska E. Rosiak I.. (2016). Colonization of germ-free mice with a mixture of three lactobacillus strains enhances the integrity of gut mucosa and ameliorates allergic sensitization. Cell. Mol. Immunol. 13, 251–262. 10.1038/cmi.2015.09, PMID: 25942514 PMC4786630

[ref100] Levkovich T. Poutahidis T. Smillie C. Varian B. J. Ibrahim Y. M. Lakritz J. R.. (2013). Probiotic bacteria induce a ‘glow of health’. PLoS One 8, e53867. 10.1371/journal.pone.0053867, PMID: 23342023 PMC3547054

[ref24] Mak R. K. Hundhausen C. Nestle F. O. (2009). Progress in understanding the immunopathogenesis of psoriasis. Actas Dermosifiliogr. 100(Suppl. 2), 2–13. 10.1016/S0001-7310(09)73372-1 20096156 PMC2957885

[ref25] Mariman R. Reefman E. Tielen F. Persoon-Deen C. van de Mark K. Worms N.. (2016). *Lactobacillus plantarum* NCIMB8826 ameliorates inflammation of colon and skin in human APOC1 transgenic mice. Benef. Microbes 7, 215–225. 10.3920/BM2015.0074, PMID: 26689228

[ref26] McFadden J. P. Baker B. S. Powles A. V. Fry L. (2009). Psoriasis and streptococci: the natural selection of psoriasis revisited. Br. J. Dermatol. 160, 929–937. 10.1111/j.1365-2133.2009.09102.x 19309365

[ref27] Naldi L. Peli L. Parazzini F. Carrel C. F. (2001). Family history of psoriasis, stressful life events, and recent infectious disease are risk factors for a first episode of acute guttate psoriasis: results of a case-control study. J. Am. Acad. Dermatol. 44, 433–438. 10.1067/mjd.2001.110876 11209111

[ref28] Noah P. W. (1990). The role of microorganisms in psoriasis. Semin. Dermatol. 9, 269–276. PMID: 2285571

[ref29] O’Neill C. A. Monteleone G. McLaughlin J. T. Paus R. (2016). The gut-skin axis in health and disease: a paradigm with therapeutic implications. Bioessays 38, 1167–1176. 10.1002/bies.201600008, PMID: 27554239

[ref30] Ramírez-Boscá A. Navarro-López V. Martínez-Andrés A. Such J. Francés R. Horga de la Parte J. (2015). Identification of bacterial dna in the peripheral blood of patients with active psoriasis. JAMA Dermatol. 151, E1–E2. 10.1001/jamadermatol.2014.5585 25760018

[ref31] Rasi A. Behzadi A. H. Davoudi S. Rafizadeh P. Honarbakhsh Y. Mehran M.. (2010). Efficacy of oral metronidazole in treatment of cutaneous and mucosal lichen planus. J. Drugs Dermatol. 9, 1186–1190. PMID: 20941941

[ref32] Reikvam D. H. Erofeev A. Sandvik A. Grcic V. Jahnsen F. L. Gaustad P.. (2011). Depletion of murine intestinal microbiota: effects on gut mucosa and epithelial gene expression. PLoS One 6:e17996. 10.1371/journal.pone.0017996, PMID: 21445311 PMC3061881

[ref33] Rutgeerts P. Hiele M. Geboes K. Peeters M. Penninckx F. Aerts R.. (1995). Controlled trial of metronidazole treatment for prevention of Crohn’s recurrence after ileal resection. Gastroenterology 108, 1617–1621. 10.1016/0016-5085(95)90121-3, PMID: 7768364

[ref34] Salem I. Ramser A. Isham N. Ghannoum M. A. (2018). The gut microbiome as a major regulator of the gut-skin axis. Front. Microbiol. 9:1459. 10.3389/fmicb.2018.01459 30042740 PMC6048199

[ref23] Schwarzer M. Hermanova P. Srutkova D. Golias J. Hudcovic T. Zwicker C. (2019). Germ-free mice exhibit mast cells with impaired functionality and gut homing and do not develop food allergy. Front. Immunol. 10:205. 10.3389/fimmu.2019.00205 30809227 PMC6379318

[ref35] Scott N. A. Andrusaite A. Andersen P. Lawson M. Alcon-Giner C. Leclaire C.. (2018). Antibiotics induce sustained dysregulation of intestinal T cell immunity by perturbing macrophage homeostasis. Sci. Transl. Med. 10. 10.1126/scitranslmed.aao4755, PMID: 30355800 PMC6548564

[ref36] Smith P. M. Howitt M. R. Panikov N. Michaud M. Gallini C. A. Bohlooly Y. M.. (2013). The microbial metabolites, short-chain fatty acids, regulate colonic Treg cell homeostasis. Science 341, 569–573. 10.1126/science.1241165, PMID: 23828891 PMC3807819

[ref37] Snel J. Heinen P. P. Blok H. J. Carman R. J. Duncan A. J. Allen P. C.. (1995). Comparison of 16S rRNA sequences of segmented filamentous bacteria isolated from mice, rats, and chickens and proposal of “Candidatus Arthromitus”. Int. J. Syst. Bacteriol. 45, 780–782. 10.1099/00207713-45-4-780, PMID: 7547299

[ref38] Steinhart A. H. Feagan B. G. Wong C. J. Vandervoort M. Mikolainis S. Croitoru K.. (2002). Combined budesonide and antibiotic therapy for active Crohn’s disease: a randomized controlled trial. Gastroenterology 123, 33–40. 10.1053/gast.2002.34225, PMID: 12105831

[ref39] Stepankova R. Powrie F. Kofronova O. Kozakova H. Hudcovic T. Hrncir T.. (2007). Segmented filamentous bacteria in a defined bacterial cocktail induce intestinal inflammation in SCID mice reconstituted with CD45RBhigh CD4^+^ T cells. Inflamm. Bowel Dis. 13, 1202–1211. 10.1002/ibd.20221, PMID: 17607724

[ref40] Tan L. Zhao S. Zhu W. Wu L. Li J. Shen M.. (2018). The *Akkermansia muciniphila* is a gut microbiota signature in psoriasis. Exp. Dermatol. 27, 144–149. 10.1111/exd.13463, PMID: 29130553

[ref41] Tett A. Pasolli E. Farina S. Truong D. T. Asnicar F. Zolfo M. (2017). Unexplored diversity and strain-level structure of the skin microbiome associated with psoriasis. NPJ Biofilms Microbiomes 3:14. 10.1038/s41522-017-0022-522 28649415 PMC5481418

[ref42] Tlaskalova-Hogenova H. Stepankova R. Kozakova H. Hudcovic T. Vannucci L. Tuckova L.. (2011). The role of gut microbiota (commensal bacteria) and the mucosal barrier in the pathogenesis of inflammatory and autoimmune diseases and cancer: contribution of germ-free and gnotobiotic animal models of human diseases. Cell. Mol. Immunol. 8, 110–120. 10.1038/cmi.2010.67, PMID: 21278760 PMC4003137

[ref43] Tsankov N. Botev-Zlatkov N. Lazarova A. Z. Kostova M. Popova L. Tonev S. (1988). Psoriasis and drugs: influence of tetracyclines on the course of psoriasis. J. Am. Acad. Dermatol. 19, 629–632. 3183091 10.1016/s0190-9622(88)70216-9

[ref44] van der Fits L. Mourits S. Voerman J. S. Kant M. Boon L. Laman J. D. (2009). Imiquimod-induced psoriasis-like skin inflammation in mice is mediated via the IL-23/IL-17 axis. J. Immunol. 182, 5836–5845. 10.4049/jimmunol.0802999 19380832

[ref45] Viaud S. Saccheri F. Mignot G. Yamazaki T. Daillere R. Hannani D.. (2013). The intestinal microbiota modulates the anticancer immune effects of cyclophosphamide. Science 342, 971–976. 10.1126/science.1240537, PMID: 24264990 PMC4048947

[ref46] Vlachos C. Gaitanis G. Katsanos K. H. Christodoulou D. K. Tsianos E. Bassukas I. D. (2016). Psoriasis and inflammatory bowel disease: links and risks. Psoriasis 6, 73–92. 10.2147/PTT.S85194ptt-6-073 29387596 PMC5683131

[ref47] Wahba-Yahav A. V. (1995). Idiopathic lichen planus: treatment with metronidazole. J. Am. Acad. Dermatol. 33, 301–302. 7622660 10.1016/0190-9622(95)90265-1

[ref48] Waldman A. Gilhar A. Duek L. Berdicevsky I. (2001). Incidence of *Candida* in psoriasis - a study on the fungal flora of psoriatic patients. Mycoses 44, 77–81. 10.1046/j.1439-0507.2001.00608.x, PMID: 11413927

[ref49] Weisenseel P. Laumbacher B. Besgen P. Ludolph-Hauser D. Herzinger T. Roecken M.. (2002). Streptococcal infection distinguishes different types of psoriasis. J. Med. Genet. 39, 767–768. 10.1136/jmg.39.10.767, PMID: 12362037 PMC1734981

[ref50] Yan D. Issa N. Afifi L. Jeon C. Chang H. W. Liao W. (2017). The role of the skin and gut microbiome in psoriatic disease. Curr. Dermatol. Rep. 6, 94–103. 10.1007/s13671-017-0178-5, PMID: 28804689 PMC5552074

[ref51] Zakostelska Z. Kverka M. Klimesova K. Rossmann P. Mrazek J. Kopecny J.. (2011). Lysate of probiotic *Lactobacillus casei* DN-114 001 ameliorates colitis by strengthening the gut barrier function and changing the gut microenvironment. PLoS One 6:e27961. 10.1371/journal.pone.0027961, PMID: 22132181 PMC3222668

[ref52] Zakostelska Z. Malkova J. Klimesova K. Rossmann P. Hornova M. Novosadova I.. (2016). Intestinal microbiota promotes psoriasis-like skin inflammation by enhancing Th17 response. PLoS One 11:e0159539. 10.1371/journal.pone.0159539, PMID: 27434104 PMC4951142

[ref53] Zanvit P. Konkel J. E. Jiao X. Kasagi S. Zhang D. Wu R. (2015). Antibiotics in neonatal life increase murine susceptibility to experimental psoriasis. Nat. Commun. 6:8424. 10.1038/ncomms9424 26416167 PMC4598725

[ref54] Zeng J. Luo S. Huang Y. Lu Q. (2017). Critical role of environmental factors in the pathogenesis of psoriasis. J. Dermatol. 44, 863–872. 10.1111/1346-8138.13806, PMID: 28349593

