# Supplementary material for: Crucial Role of Microbiota in Experimental Psoriasis Revealed by a Gnotobiotic Mouse Model
Source: Front Microbiol. 2019 Feb 21;10:236. doi: 10.3389/fmicb.2019.00236 (PMC6394148; doi:10.3389/fmicb.2019.00236)
Supplement: Supplementary file 1 [file Table_1.docx]

List of abbreviations

ANOVA analysis of variance

ATB antibiotic

CFU colony forming units

COL colistin

CV conventional

Cxcl C-X-C motif chemokine ligand 1

Eef2 eukaryotic translation elongation factor 2

GF germ free

IBD inflammatory bowel disease

IISI imiquimod-induced skin inflammation

Il interleukin

IMQ imiquimod

ING inguinal lymph nodes

LEfSe linear discriminant analysis effect size

LP *Lactobacillus plantarum* WCFS1

MET metronidazole

MIX mixture of antimicrobials

Nfkbiz nuclear factor of kappa light polypeptide gene enhancer in B-cells inhibitor, zeta

PASI psoriasis area and severity index

QIIME quantitative insights into microbial ecology

RORγt rar-related orphan receptor gamma

SD standard deviation

SEM standard error of the mean

SFB segmented filamentous bacteria

SPL spleen

STR streptomycin

Th17 T-helper 17 cell

VAN vancomycin

V3-V4 variable regions of 16S rRNA

16S component of 30S small subunit of prokaryotic ribosomal RNA
