# Supplementary material for: Crucial Role of Microbiota in Experimental Psoriasis Revealed by a Gnotobiotic Mouse Model
Source: Front Microbiol. 2019 Feb 21;10:236. doi: 10.3389/fmicb.2019.00236 (PMC6394148; doi:10.3389/fmicb.2019.00236)
Supplement: Supplementary file 2 [file Table_2.docx]

Supplementary information

**Crucial role of microbiota in experimental psoriasis revealed by a gnotobiotic mouse model**

Zuzana Stehlikova^1,2^, Klara Kostovcikova^1,3^, Miloslav Kverka^1,4^, Pavel Rossmann^1^, Jiri Dvorak^1^, Iva Novosadova^1^, Martin Kostovcik^1,5^, Stepan Coufal^1^, Dagmar Srutkova^6^, Petra Prochazkova^1^,Tomas Hudcovic^6^, Hana Kozakova^6^, Renata Stepankova^6^, Filip Rob^7^, Katerina Juzlova^7^, Jana Hercogova^7^, Helena Tlaskalova-Hogenova^1^ and Zuzana Jiraskova Zakostelska^1*^

^1^Institute of Microbiology of the Czech Academy of Sciences, v.v.i., Prague, Czech Republic; ^2^Charles University, First Faculty of Medicine, Prague, Czech Republic; ^3^Institute of Molecular Genetics of the Czech Academy of Sciences, v.v.i., Prague, Czech Republic; ^4^Institute of Experimental Medicine of the Czech Academy of Sciences, v.v.i., Prague, Czech Republic; ^5^BIOCEV, Institute of Microbiology, Czech Academy of Sciences, Vestec, Czech Republic; ^6^Institute of Microbiology of the Czech Academy of Sciences, v.v.i., Novy Hrádek, Czech Republic; ^7^Charles University, Second Faculty of Medicine, Department of Dermatology and Bulovka Hospital, Prague, Czech Republic.

*** Correspondence:** Zuzana Jiraskova Zakostelska: zakostelska@biomed.cas.cz

Supplementary material and methods

Quantification of the total amount of bacteria, *Lactobacillus* sp. and segmented filamentous bacteria in conventional mice before and after antibiotic treatment

The abundances of specific intestinal bacterial groups were measured by qPCR (CFX96 Touch, Bio-Rad) using a group-specific 16S rRNA gene or species-specific primer sets (total bacteria, *Lactobacillus* sp., segmented filamentous bacteria (SFB), *A. muciniphila*). The amplifications were performed in 25 μl reaction mixtures (iQ™ SYBR^®^ Green Supermix, Bio-Rad) containing the same amount of gDNA template in each sample (40 ng). The names, sequences and origins of primers are shown in the table below (Byun et al., 2004; Collado et al., 2007; Denman and McSweeney, 2006; Snel et al., 1995). Cycling parameters were as follows: 4 min at 94 °C, 35 cycles of 10 s at 94 °C, 25 s at 60 °C, and 35 s at 72 °C, and a final extension for 7 min at 72 °C. Amplifications were performed in duplicates.

The PCR fragments of corresponding genes were ligated into the pCR2.1-TOPO cloning vector (Life Technologies) with subsequent transformation into chemically competent *E. coli* cells (Neb 5-alpha *E. coli*, Bio Labs). The specificity of the purified plasmids was verified by sequencing. To obtain their linear form, plasmids were cut with BamH1 restriction endonuclease (37 °C, 12 hours). Purified plasmids containing the corresponding 16S rRNA gene inserts were used as the standard to validate the absolute copy number of each sample in 10-fold dilution series.

PCR primers used in quantification.

| **Name** | **Sequence (5´- 3´)** | **Target** | **References** |
| --- | --- | --- | --- |
| BAC | CGGCAACGAGCGCAACCC | universal bacterial 16S rRNA | Denman and McSweeney 2006 |
| BAC | CCATTGTAGCACGTGTGTAGCC |  |  |
| LactoF | TGGAAACAGRTGCTAATACCG | Lactobacilli 16S rRNA | Byun et al. 2004 |
| LactoR | GTCCATTGTGGAAGATTCCC |  |  |
| SFB736 | GACGCTGAGGCATGAGAGCAT | SFB 16S rRNA | Snel et al. 1995 |
| SFB844 | GACGGCACGGATTGTTATTCA |  |  |
| AM1129F | CAGCACGTGAAGGTGGGGAC | Akkermansia muciniphila 16S rRNA | Collado et al. 2007 |
| AM1437R | CCTTGCGGTTGGCTTCAGAT |  |  |

Statistical analysis of data

Analyses of changes in the total bacteria number and amounts of *Lactobacillus sp.,* SFB or *A. muciniphila* were determined using a two-way analysis of variance, with a *p-*value of <0.05 being considered significant.

**Supplementary figures**

Supplementary figure 1 (related to MATERIAL AND METHODS: Flow cytometry analysis of induced immune response)


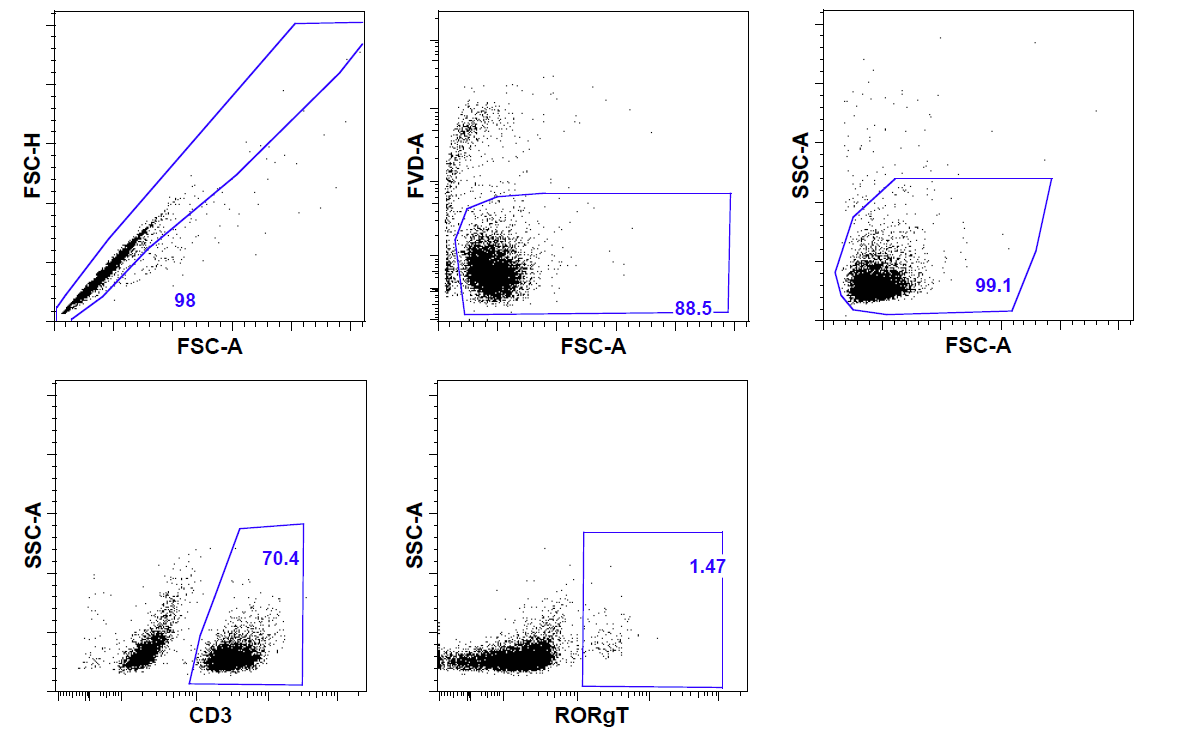


**SUPPLEMENTARY FIGURE 1** Gating strategy for CD3^+^ RORγt^+^ analysis by flow cytometry. Cells from the spleen or inguinal lymph nodes were isolated and analyzed for surface CD3 and intracellular RORγt by flow cytometry. Cells were first gated for live cells and CD3^+^, and subsequently for RORγt^+^ cells as shown in the example of gating strategy. To normalize the data between experiments we expressed controls as a 100% of live CD3^+^ RORγt^+^ cells.

Supplementary figure 2 (related to Figure 1)


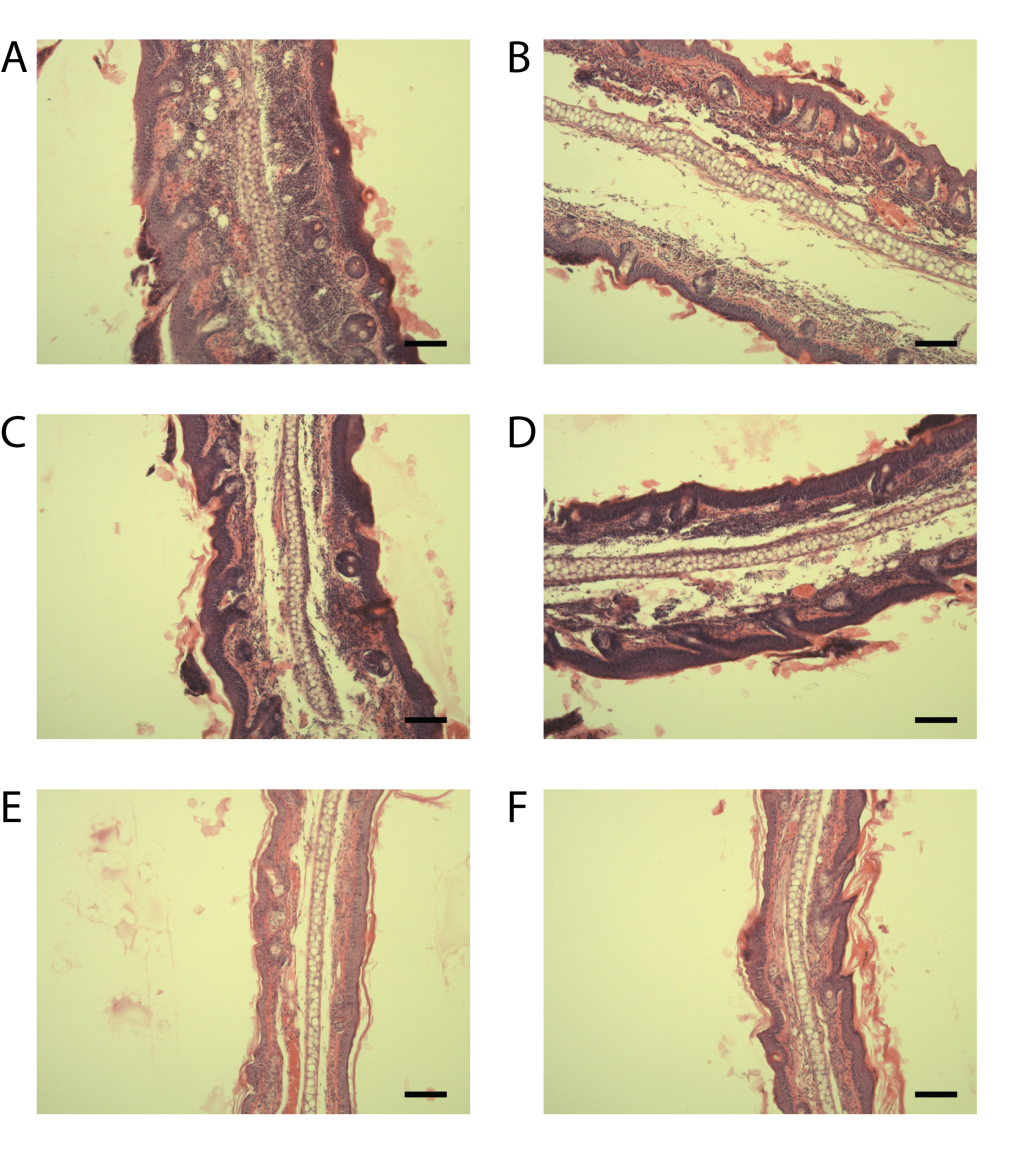


**SUPPLEMENTARY FIGURE 2** Representative histological sections of H&E-stained ears of BALB/c mice (scale bar 100 μm). **(A)** Control mice, **(B)** colistin-treated mice, **(C)** vancomycin-treated mice, **(D)** streptomycin-treated mice, **(E)** metronidazol-treated mice, and **(F)** mice treated with mix of antibiotics.

Supplementary figure 3 (related to Figure 1)


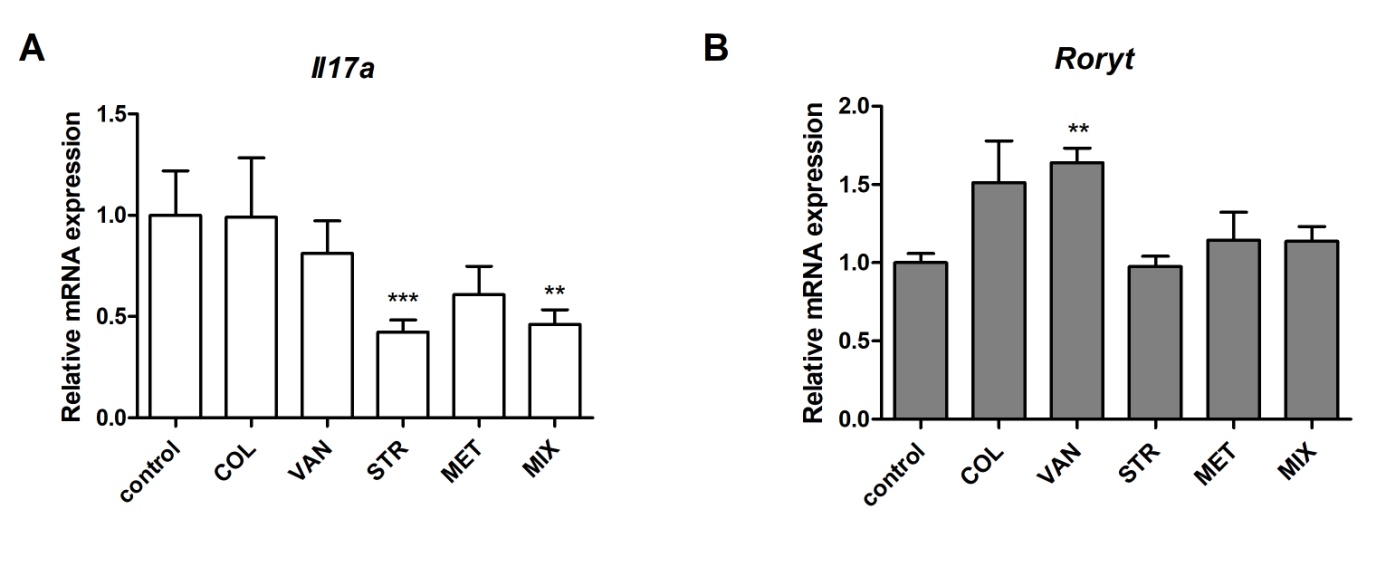


**SUPPLEMENTARY FIGURE 3.** Treatment with a single antibiotic or antibiotic mixture changes the susceptibility to IMQ-induced skin inflammation in BALB/c mice. Quantitative PCR analysis of mRNA expression of *Il17a* **(A)** and *Rorγt* **(B)** in the skin. Data are normalized to the expression of the Elongation factor 2 (*eEF2*) as a reference gene and are presented as mean ± SEM. The graphs show the results of one representative experiment out of two independent experiments (n = 4-5 mice per group). We used two-tailed t-test to determine the statistical significance between the groups. *p < 0.05, **p < 0.01,***p < 0.001.

Supplementary figure 4 (related to Figure 3)


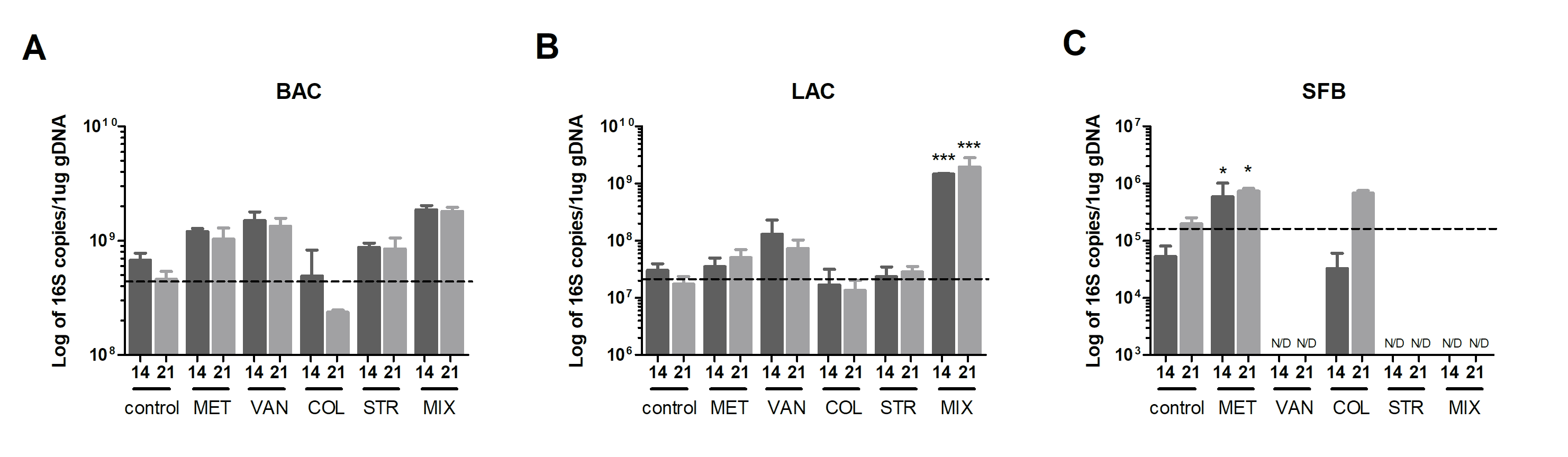


**SUPPLEMENTARY FIGURE 4** Quantification of the total amount of bacteria, *Lactobacillus*sp. and segmented filamentous bacteria in conventional mice before and after antibiotic treatment. The total amount of bacteria and abundances of specific intestinal bacterial groups were measured by quantitative real-time PCR. **(A)** The total amount of bacteria (BAC). **(B)** The abundance of *Lactobacillus* sp. (LAC) **(C)** The abundance of segmented filamentous bacteria (SFB). Data are presented as mean ± SEM (n = 3-8 in each group). Significant difference from the beginning of the experiment (Day 0) is denoted by *p < 0.05, **p < 0.01, ***p < 0.001. The dashed line represents the amount of bacteria on Day 0. Statistical significance in graph was determined by two-way ANOVA.

Supplementary Table 1 (related to Figure 3)

**SUPPLEMENTARY TABLE 1.** Distinctive skin microbial taxa distinguished by LEfSe between control mice and mice treated with MET and MIX on Day 14 and Day 21.

| **Distinctive composition of mouse skin microbiota determined using LEfSe** | | | |
| --- | --- | --- | --- |
| **day** | **control** | **MIX** | **MET** |
| **14** | g_*Streptococcus* | g_*Lactobacillus* | s_*Escherichia coli* |
|  | o_Bacteroidales (f_S24_7) | c_Bacilli | s_*Paenibacillus barengoltzii* |
|  | c_Mollicutes | g_*Enterobacter* | g_*Bacteroides* |
|  | c_Clostridia | g_*Turicibacter* | s_*Bacteroides acidifaciens* |
|  | f_Lachnospiraceae | g_*Stenotrophomonas* | g_*Enterococcus* |
|  | o_Neisseriales | g_*Allobaculum* | s_*Bifidobacterium pseudolongum* |
|  | g_*Helicobacter* | g_*Adlercreutzia* | p_Actinobacteria |
|  | o_Pasteurellales | g_*Serratia* | g_*Parabacteroides* |
|  | o_Campylobacterales | g_*Pseudomonas* | f_Porphyromonadaceae |
|  | g_*Acinetobacter* | s_*Clostridium celatum* | o_Bacteroidales (f_S24_7) |
|  | g_*Prevotella* | o_Coriobacteriia | g_*Sutterella* |
|  | s_*Actinobacillus muris* | g_*Sphingobium* | s_*Klebsiella oxytoca* |
|  | s_*Akkermansia muciniphila* | g_*Erwinia* | g_*Coprobacillus* |
|  | **control** | **MIX** | **MET** |
| **21** | g_*Staphylococcus* | g_*Lactobacillus* | g_*Enterococcus* |
|  | o_Bacillales | o_Bacteroidales (f_S24_7) | s_*Bifidobacterium pseudolongum* |
|  |  | g_*Turicibacter* | g_*Staphylococcus*_other |
|  |  | g_*Adlercreutzia* | f_Erysipelotrichaceae |

Abbreviations : f_family, c_class, o_order, g_genus, s_species

Supplementary Table 2 (related to Figure 3)

**SUPPLEMENTARY TABLE 2.** Distinctive microbial taxa distinguished by LEfSe analysis between control mice and mice treated with MET and MIX on Day 14 and Day 21 in the intestine.

| **Distinctive composition of mouse fecal microbiota using LEfSe** | | | |
| --- | --- | --- | --- |
| **day** | **control** | **MIX** | **MET** |
| **14** | o_Bacteroidales (f_S24_7) | g_*Lactobacillus* | s_*Bacteroides acidifaciens* |
|  | o_Clostridiales | s_*Staphylococcus sciuri* | s_*Bifidobacterium pseudolongum* |
|  | g_*Prevotella* | g_*Staphylococcus*_other | f_Enterococcaceae |
|  | f_Lachnospiraceae | g_*Comamonas* | g_*Paenibacillus* |
|  | f_Ruminococcaceae |  | g_*Parabacteroides* |
|  | g_*Bacteroides* |  | g_*Sutterella* |
|  | f_Rikenellaceae |  | s_*Klebsiella oxytoca* |
|  | s_*Akkermansia muciniphila* |  | c_Coriobacteriia |
|  | s_*Lactobacillus reuteri* |  | g_*Enterococcus* |
|  | g_*Oscillospira* |  | g_*Allobaculum* |
|  | o_Campylobacterales |  | s_*Lactobacillus acidipiscis* |
|  | g_*Helicobacter* |  | s_*Coprobacillus cateniformis* |
|  | g_*Coprococcus* |  | s_*Parabacteroides distasonis* |
|  | g_*Anaerotruncus* |  |  |
|  | f_Moraxellaceae |  |  |
|  | s_*Alistipes massiliensis* |  |  |
|  | s_*Ruminococcus gnavus* |  |  |
|  | f_Microbacteriaceae |  |  |
|  | g_*Adlercreutzia* |  |  |
|  | g_*Clostridium* |  |  |
|  | g_*Trabulsiella* |  |  |
|  | c_TM7 |  |  |
|  | g_*Odoribacter* |  |  |
|  | s_*Butyricicoccus pullicaecorum* |  |  |
|  | g_*Rikenella* |  |  |
|  | s_*Paenibacillus lentimorbus* |  |  |
|  | s_*Enterobacter gergoviae* |  |  |
|  | s_*Parabacteroides gordonii* |  |  |
|  | s_*Lactobacillus vaginalis* |  |  |
|  | **control** | **MIX** | **MET** |
| **21** | o_Clostridiales | g_*Lactobacillus* | c_Actinobacteria |
|  | f_Lachnospiraceae | s_*Escherichia coli* | s_*Bifidobacterium pseudolongum* |
|  | o_Bacteroidales (f_S24_7) |  | s_*Bacteroides acidifaciens* |
|  | g_*Oscillospira* |  | f_Erysipelotrichaceae |
|  | s_*Parabacteroides gordonii* |  | f_Enterobacteriaceae |
|  | s_*Ruminococcus gnavus* |  | g_*Sutterella* |
|  | g_*Bacteroides* |  | s_*Parabacteroides distasonis* |
|  | g_*Streptococcus* |  | f_Coriobacteriaceae |
|  | o_Campylobacterales |  | s_*Actinobacillus muris* |
|  | g_*Helicobacter* |  | g_*Enterococcus* |
|  | s_*Lactobacillus reuteri* |  |  |
|  | g_*Prevotella* |  |  |
|  | g_*Adlercreutzia* |  |  |
|  | g_*Staphylococcus* |  |  |
|  | s_*Akkermansia muciniphila* |  |  |
|  | g_*Odoribacter* |  |  |
|  | s_*Mucispirillum schaedleri* |  |  |
|  | s_*Butyricicoccus pullicaecorum* |  |  |
|  | s_*Alistipes massiliensis* |  |  |
|  | g_*Anaeroplasma* |  |  |
|  | s_*Oscillospira guiliermondii* |  |  |

Abbreviations : f_family, c_class, o_order, g_genus, s_species

Supplementary Figure 5

**SUPPLEMENTARY FIGURE 6.** Quantification of the total amount of *Akkermansia muciniphila* in the intestine of IMQ-treated and control mice. The abundance of *Akkermansia muciniphila* was measured by quantitative real-time PCR. The mice were treated daily for 6 consecutive days with IMQ or control cream (control) on their shaved back and left ear. The amounts of bacteria before and after treatment (Day 1 and Day 7) are shown. Statistical significance was determined by two-way ANOVA.

**
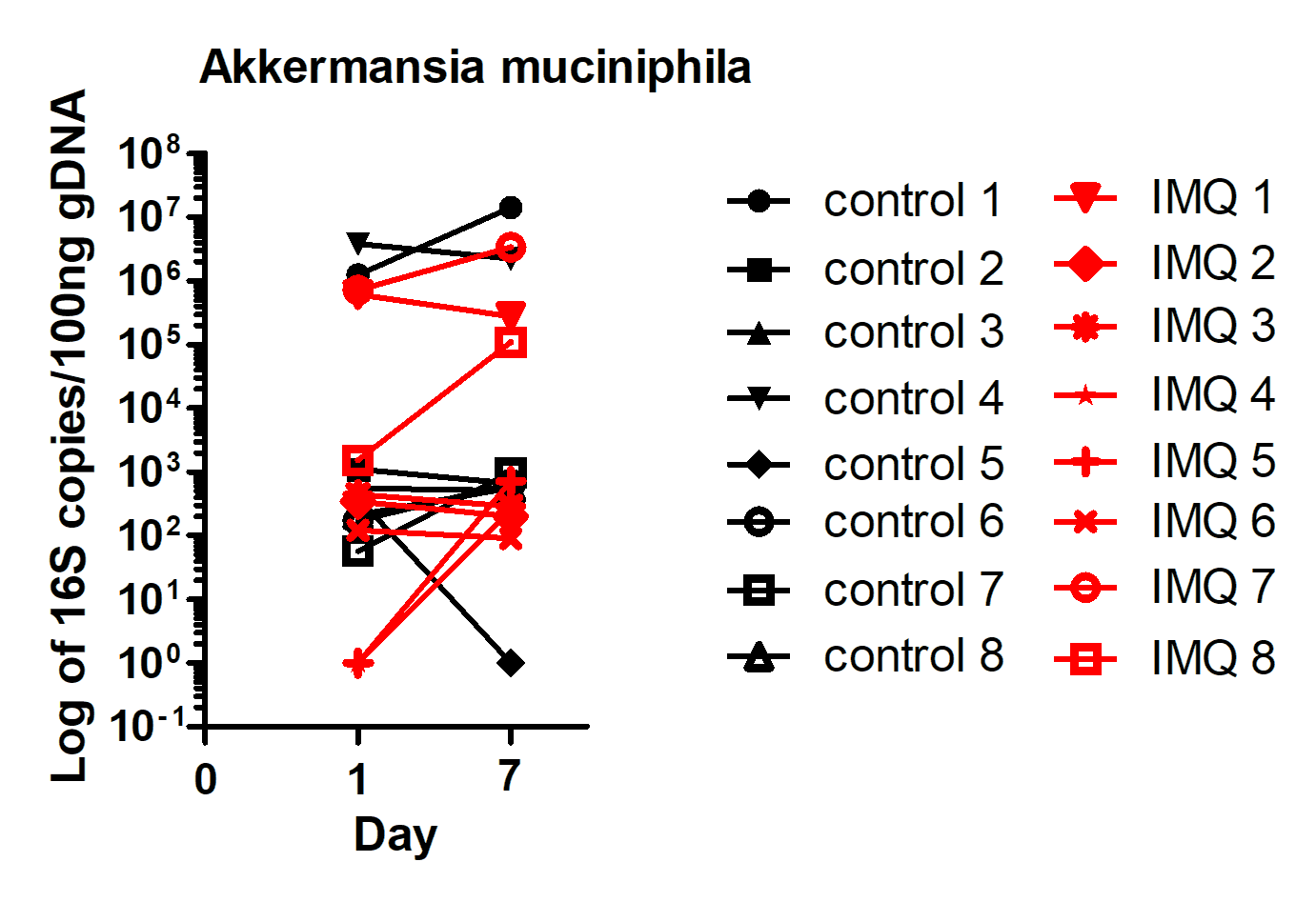
**

**References**

Byun, R., Nadkarni, M.A., Chhour, K.L., Martin, F.E., Jacques, N.A., and Hunter, N. (2004). Quantitative analysis of diverse Lactobacillus species present in advanced dental caries. *J Clin Microbiol* 42, 3128-3136. doi: 10.1128/JCM.42.7.3128-3136.2004

Collado, M.C., Derrien, M., Isolauri, E., de Vos, W.M., and Salminen, S. (2007). Intestinal integrity and Akkermansia muciniphila, a mucin-degrading member of the intestinal microbiota present in infants, adults, and the elderly. *Appl Environ Microbiol* 73, 7767-7770. doi: AEM.01477-07

Denman, S.E., and McSweeney, C.S. (2006). Development of a real-time PCR assay for monitoring anaerobic fungal and cellulolytic bacterial populations within the rumen. *FEMS Microbiol Ecol* 58, 572-582. doi: FEM190

Snel, J., Heinen, P.P., Blok, H.J., Carman, R.J., Duncan, A.J., Allen, P.C., et al. (1995). Comparison of 16S rRNA sequences of segmented filamentous bacteria isolated from mice, rats, and chickens and proposal of "Candidatus Arthromitus". *Int J Syst Bacteriol* 45, 780-782. doi: 10.1099/00207713-45-4-780
